# Custom-made 3D-printed X-ray shield for tumor-specific irradiation of xenograft mice

**DOI:** 10.1186/s41205-025-00264-z

**Published:** 2025-04-07

**Authors:** Markus Lechner, Anna Kolz, Kristina Herre, Dana Matzek, Adrian Schomburg, Bastian Popper

**Affiliations:** 1 Eisbach Bio GmbH, Am Klopferspitz 19, 82152 Planegg-Martinsried, Germany; 2 Biomedical Center, Core Facility Animal Models, Faculty of Medicine, Ludwig- Maximilians-Universität München, Großhaderner Straße 9, 82152 Planegg-Martinsried, Germany; 3 Biomedical Center, Department of Physiological Chemistry, Faculty of Medicine, Ludwig-Maximilians-Universität München, Großhaderner Straße 9, 82152 Planegg- Martinsried, Germany

**Keywords:** Tungsten, Tumor, Radiation

## Abstract

**Background:**

Xenograft mouse models play an important role in preclinical cancer research, particularly in the development of new therapeutics. To test the efficacy of a combination therapy consisting of radiation and new drug candidates, it is crucial that only the tumor area is irradiated, while other parts of the body are shielded. In this study, a 3D-printed radiopaque back shield was designed for tumor-specific irradiation and evaluated in a xenograft mouse model.

**Methods:**

Different radiopaque materials were initially tested for their shielding properties using the Multirad 225 X-ray irradiator and the most suitable material was used for printing a back shield with a tumor site-specific opening of the cover. Tumor bearing mice were irradiated four times with a dose of 3.5 Gy. To evaluate proper body shielding, blood samples, spleens and bone marrow were examined at the end of the experiment.

**Results:**

A tungsten filament was identified to be most efficient for shielding and used to 3D print a pie-slice-shaped back shield with a tumor-site specific opening, while polylactic acid was used to print a scaffold that ensured proper positioning of the shield. The simple design allowed cost-efficient and fast 3D printing, easy handling and individual modifications of the tumor site openings. In terms of animal safety, the product provided sufficient shielding in the low-dose irradiation protocols of xenograft mice.

**Conclusion:**

The custom-designed 3D-printed tungsten back shields provide proper shielding of the animals body and allow for subcutaneous tumor irradiation under standardized conditions.

## Introduction

Cancer research leveraging murine models, particularly through the implementation of xenograft tumors, has provided invaluable insights into tumor biology, metastasis, and potential therapeutic strategies [1]. Murine cancer models have been improved over the last decades to best reflect the disease in humans, a prerequisite to develop new treatment methods and to establish improved therapy strategies [2]. The development of patient-derived xenograft mouse models by injection of tumor tissue or cells subcutaneously has further advanced the knowledge of intratumor characteristics and allowed tumor site-specific treatment and manipulation [1]. Irradiation of solid tumors and co-treatment with a variety of other techniques, e.g. chemotherapy, is established for many tumor entities in preclinical research [3]. Precise and accurate irradiation of those tumor sites in xenograft mouse models is essential to avoid side effects such as bone marrow suppression and hematopoietic injury resulting from total body radiation [4–6]. Previously, lead shielding and custom polyacrylic containers were used to achieve this. The purpose of this study was to develop a 3D printable, custom-designed back shield that allows repeated tumor site-specific irradiation of xenograft mouse models in a cabinet irradiator while protecting the rest of the animal’s body. The simple design and the cost-effective selection of materials for 3D printing should enable facilities to manufacture customized products based on our template to further improve animals’ safety in preclinical research.

## Background

In order to enable tumor site-specific irradiation for small rodents, the irradiation devices are specially adapted to the size of the animals and tumors, and are usually designed for the simultaneous irradiation of several animals in order to ensure the standardization of treatment measures and improve the robustness of data [7]. The use of completely closed shields allows the simultaneous exposure of tumor-bearing control animals in the same apparatus under the same test conditions. Further, the rest of the animal’s body must be shielded by a radiation-proof material that can be adapted to the size of the animal and the shape of the tumor [6–8]. Advances in 3D printing technology and availability of metal-containing filaments offers unique advantages to custom-make shields best suited for specific irradiation devices, and more important the animal’s anatomy as well as the tumor-side and growth-specific requirements.

## Materials and methods

### Model development and testing

The Bambu Lab P1S printer (Bambulab, Shenzhen, China) was used for all prints using 0.4 nozzle for PLA and hardened steel hotend 0.6 nozzle for all metal filaments.

For shielding capacity tests, 60 mm diameter discs were printed with 5 mm strength, resembling the maximum metal layer strength of the final cover. These discs fully covered the built-in dosimeter of the Multirad 225 device to measure remaining radiation values through the print at different x-ray settings.

Tested filaments: Prusamet PETG Tungsten 75% (Prusa Polymers, Prague, Czech Republic), Metalfil Classic Copper and Bronze (Formfutura, Nijmegen, Netherlands), Aluminum (Virtual Foundry, Stoughton, US), Steel Ultrafuse 316 L (BASF 3D Printing Solutions GmbH, Heidelberg, Germany), Polylactic acid (PLA + 175B1, eSUN, Shenzen, China).

### 3D printing of shields

Pie-shaped base molds are printed with PLA + at 30% infill rate, using roughly 50 g of material per piece (0.90 € per unit at 18 €/kg filament). Tungsten shields were printed using Prusament PETG Tungsten with 100% rectilinear infill pattern, resulting in shields of roughly 160 g per piece (40 € per unit at 250 €/kg filament).

### Animal model

8-week-old female NMRI outbred mice (Crl: NMRI (Han), Charles river, Germany) were allocated randomly to 2 groups (*n* = 8 animals/group) and housed in groups of 4 individuals per cage. For the xenograft control and xenograft irradiation groups, the mice were subjected to tumor cell injection followed by irradiation experiments (Fig. 2). Additionally, 15 NMRI outbred mice were processed as controls and dissected either on day 1 or day 14.

The trial was approved by the government of Upper Bavaria (Az. 02-21-171). Housing of laboratory mice was in accordance with European and German animal welfare legislations (5.1–231 5682/LMU/BMC/CAM). Room temperature and relative humidity ranged from 20 to 22 °C and 45–55%, respectively. The light cycle was adjusted to 12 h light:12 h dark period. Room air was exchanged 11 times per hour and filtered with HEPA-systems. All mice were housed in individually ventilated cages (EURO Typ II long, Tecniplast, Germany) under specified-pathogen-free conditions. Hygiene monitoring was performed every three months based on the recommendations of the FELASA-14 working group. All animals had free access to water and food (irradiated, 10 mm pellet; 1314P, Altromin, Germany). The cages were equipped with nesting material (5 × 5 cm, Nestlet, Datesand, UK), a red corner house (Tecniplast, Germany) and a rodent play tunnel (7.5 × 3.0 cm, Datesand, UK). Soiled bedding (Animal bedding fine, LTE-E-002, Abedd, Austria) was replaced every 7 days.

### Tumor cell injection and randomization

Syngeneic murine tumor cells diluted in 100 µl 1:1 PBS and Matrigel (Thermo Fisher, Germany) were subcutaneously injected into the right flank of the animals on day 1 (Fig. 2). Randomization of mice took place on day 7. Three animals were removed from the xenograft control group and one animal from the xenograft irradiation group after the tumor had not reached the required size at the start of the trial.

### Irradiation protocol

The MultiRad 225 (Faxitron, Germany) X-ray irradiation system was used for all experiments. Before each radiation step, the system conducted a self-examination, preheating and dose correction based on the internal software. The distance from the source to the skin was standardized at 58 cm, the thickness of the copper filter was 0.5 mm and the energy voltage was set to 120 kV. The current was adjusted according to the dose rate measured by the internal dose monitor. Mice were anesthetized by intraperitoneal injection of Ketamin (80 mg/kg) and Xylazine (16 mg/kg). Irradiation was conducted on four time points between day 7 and day 14 (Fig. 2) with a single-dose of 3.5 Gy (120 kV and 20 mA).

### Sample collection and histology

Blood samples were taken from deeply anesthetized animals via heart puncture at day 1 and day 14. Samples were analyzed using the automated Element HT5 (Scilvet, Germany) system. At the end of the experiment, animals were killed by cervical dislocation and ultimately dissected to harvest spleens and femur bones for histological evaluation. Tissues were formalin-fixed, paraffin-embedded and stained with hematoxylin and eosin (H&E). Images were acquired using a Zeiss Axiovert 5 microscope connected to a Axiocam 205 camera system.

### Statistics

Statistics were conducted using GraphPad Prism version 5.04 (GraphPad Software, San Diego, CA, USA). Kruskal–Wallis (K–W) test followed by Dunn’s multiple comparison (non-parametric data) was used to determine the *p*-values. Data are shown as mean and SEM values if not stated otherwise. The significance level was set to *α* < 0.05.

## Results

First, six different filaments were tested regarding their shielding capacity in three experimental set-ups using the built-in dosimeter of the Multirad 225 irradiation chamber. For this, discs of 60 mm diameter were printed from each material to cover the dosimeter and the remaining radiation strength was recorded as Gy/min at each setting.

The tungsten filament showed the best shielding properties (Table 1) and was chosen to 3D print a back shield for tumor-site specific irradiation of xenograft mice and respective controls.

**Table 1:** Physical X-ray shielding properties of different filaments. Values are the measured remaining radiation values detected below the printed discs in Gy/min

Setting	ADC, 200 kV, 20 mA, Shelf position 2, Co 0.5 mm filter	ADC, 160 kV, 20 mA, Shelf position 2, Co 0.5 mm filter	160mV, 20 mA, Shelf position 2 no filter
**Shield material**			
No shield	0.951	0.55	10.225
Polylactic acid (PLA)	0.926	-	-
Copper	0.407	0.193	0.064
Bronze	0.379	0.178	0.051
Tungsten	0.018	0.007	0.008
Aluminum	0.892	0.515	1.555
Steel	0.402	0.193	0.064

# The values without filter may deviate from the measurements with filter, as the device has an internal sensitivity setting for the selected filter. The values can only be compared within each column

**Fig. 1:**
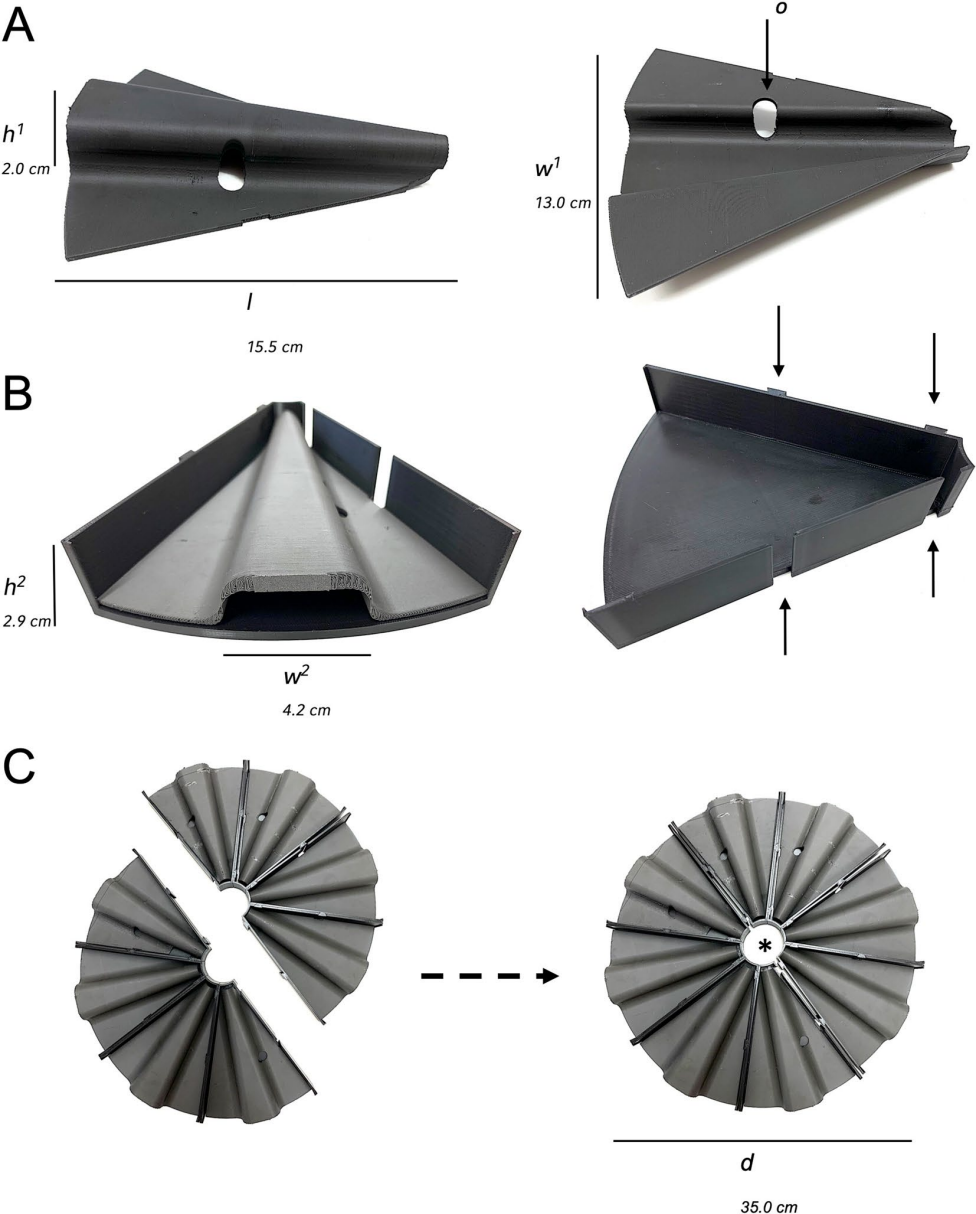
3D-printed back shield made of tungsten filament. **A**. Left panel, site view of the shield and right panel, view from below showing one-sided opening for tumor placement. **B**. Back shield placed in 3D-printed pie-shaped mold made of plastic filament. Site view of pie-shaped mold with two toothing holders on each site (arrows). **C**. “Cake and half cake” assembly of the pie-shaped molds and back shields with recessed area for the dosimeter (*) in the middle. *d = diameter*,* h*^*1*^ *= height of back shield back bone*,* h*^*2*^ *= height of the molds*,* l = total length*,* w*^*1*^ *= total weight*,* w*^*2*^ *= weight of the shields back bone*

The shape of the shield and mold was designed using the Shapr3D software to cover the entire animal’s body while the animal was restrained in a forwarded position with the nose towards the tip of the pie-slice-shaped mold. Therefore, a hole of 1.1 cm diameter was cut out to allow tumor site-specific irradiation (Fig. 1A).

**Fig. 2:**
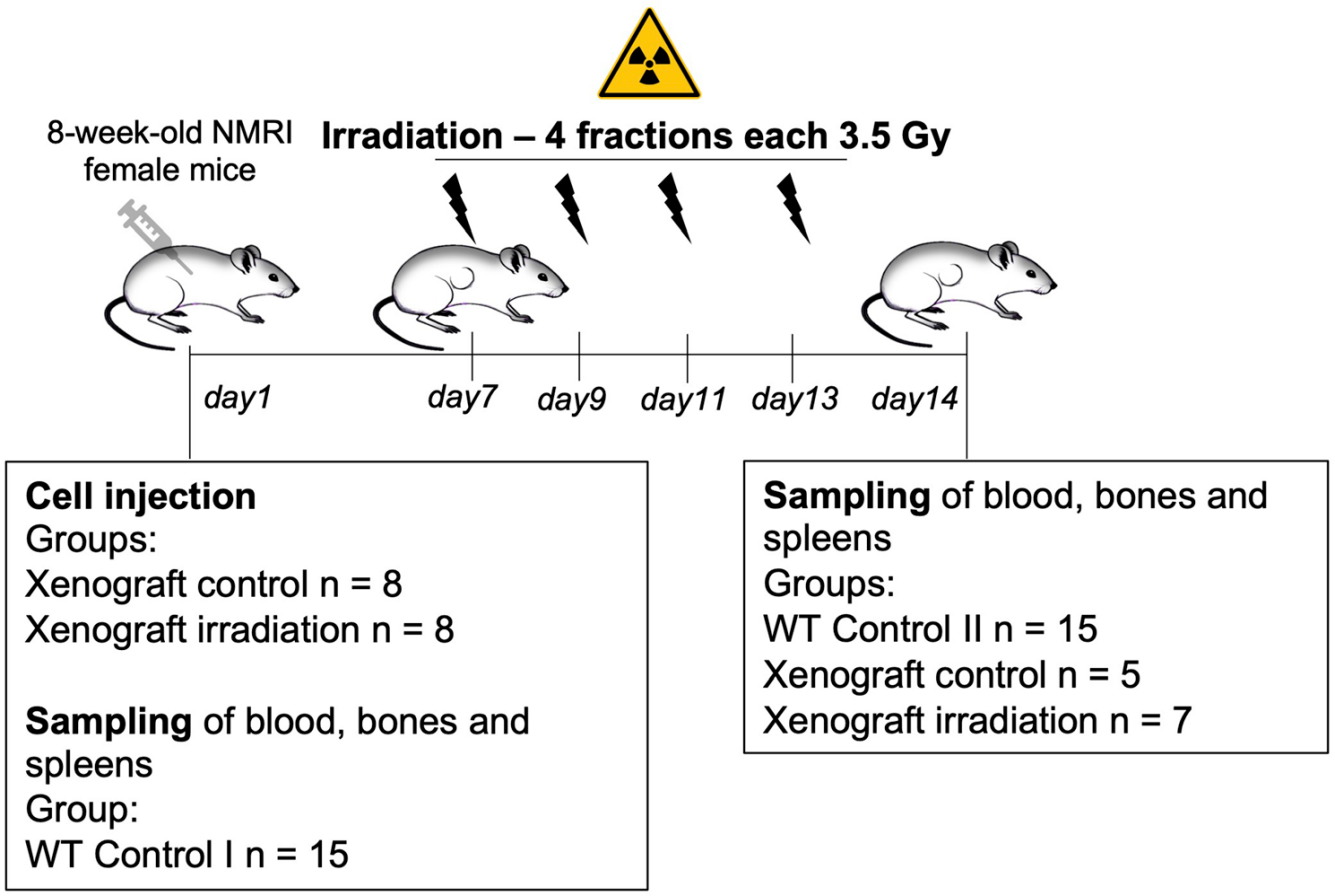
Experimental procedure and flow diagram of this study

**Fig. 3:**
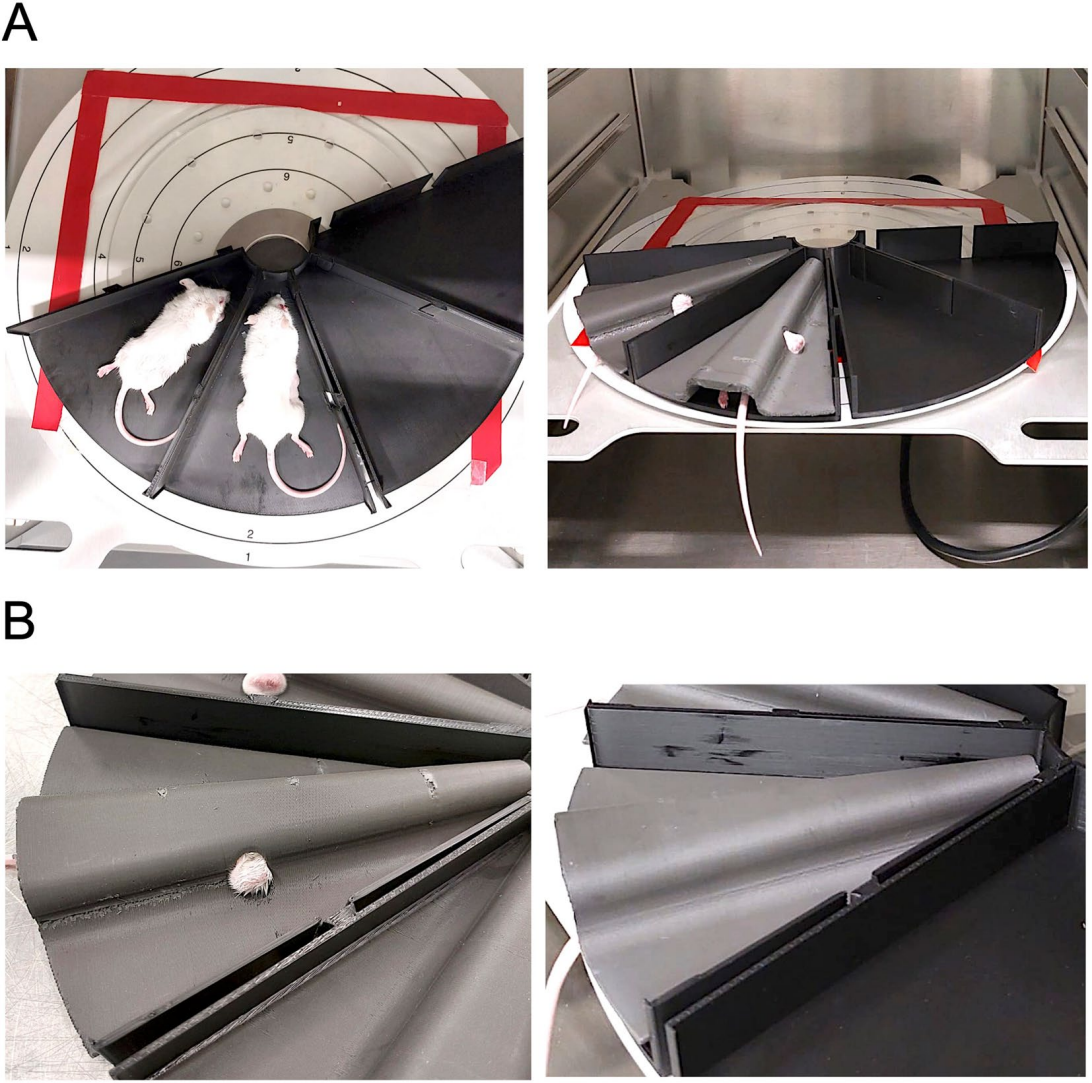
Irradiation setup. **A**. 3D printed plastic mold and back shield made of tungsten filament with two anesthetized xenograft animals in the cabinet irradiator. **B**. 3D printed pie-shaped X-ray shields with tumor site opening (left) used in the xenograft irradiation group and closed shield (right) used in the xenograft control group

The custom-designed back shields were placed in PLA-printed pie-shaped molds (Fig. 2B) that could easily be assembled in a circular manner by connecting the toothing holders on each side of the mold to ensure proper stability even on a rotating platform (Fig. 2C). A cake consisting of 8 restrainers fitted the Multirad 225 irradiation chamber while the dosimeter opening remained unshielded (Fig. 2C). The covers laid directly on the back of the animals; however, the weight of the cover was carried by the wing plates on the sides (Figs. 1B and 3A). The narrow opening at the front of the cover allowed the animals sufficient ventilation during the experimental process. Furthermore, we examined the manufactured components with regard to their handling and shielding capabilities in a xenograft mouse model (Fig. 2). We irradiated two groups of tumor mice with either tumor-site specific back shield openings (Fig. 3B, left image) or completely closed back shields as controls (Fig. 3B, right image). Mice were anesthetized and placed in the pie-shaped molds before they were transferred to the irradiation camber (Fig. 3A/B).

**Fig. 4:**
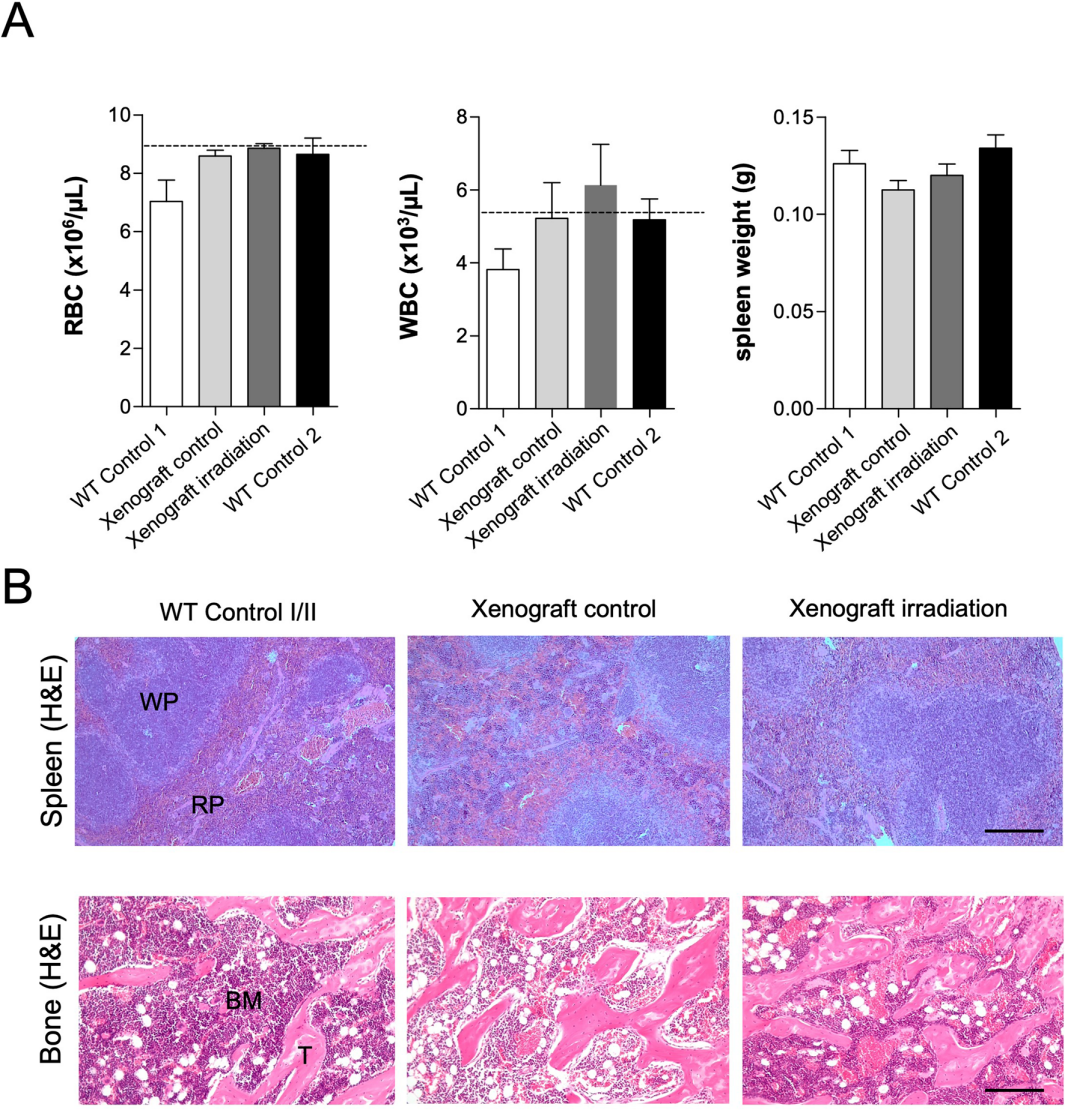
Blood and tissue analysis. Hematology of red (RBC) and white blood cells (WBC) and spleen weights at the end of the experimental setup. **B**. Histological evaluation of H&E-stained sections of the white (WP) and red splenic pulp (RP), bone marrow (BM) and femoral trabeculae (T) at day 14. Data are depicted as mean + SEM. Scale bar: 100 μm

Control animals were examined before the start of X-ray exposure as well as at the end of the test series in order to evaluate the effects of irradiation in non-tumor-bearing and xenograft mice. Blood samples of irradiated mice did not show evidence of radiation-induced damage (Fig. 4A) since there is no statistically significant difference in either red blood cells (RBC) or white blood cells (WBC) among the groups tested. For ethical reasons, we did not expose control mice to unshielded irradiation. The measured total numbers of red blood cells in the two WT control groups, day 1 and day 14, were slightly lower than those for the irradiation groups (xenograft control/irradiation). The mean values of the analyzer used as a basis for the species were not exceeded and are comparable to untreated reference animals of the same species for the xenograft control/irradiation groups (Fig. 4A, left panel). The total number of WBC in the control groups was also below or within the mean value range of untreated reference animals. The control group with irradiation and completely covered back plate (xenograft control) showed no decrease in WBC numbers. The irradiation group with lateral opening of the tumor area (xenograft irradiation) showed slightly increased but not significantly changed values for WBCs compared to the other groups investigated (Fig. 4A middle panel), probably attributable to an immune reaction against the syngeneic tumor. However, we found a slight but non-significant decrease in spleen weights in both the xenograft control and xenograft irradiation cohort compared to WT control animals (Fig. 4A right panel). Next, we performed a histological analysis of spleens and bone to investigate radiation-induced damage to these organs. The morphology of the red and white splenic pulp appeared normal in hematoxylin and eosin-stained paraffin sections among all groups tested (Fig. 4B, upper panel). Further, we analyzed the bone marrow and trabeculae structure of bones collected from WT control and xenograft control/irradiation animals at the end of the experiment. Bone marrow cells of xenograft control and irradiation cohorts showed no difference compared to the WT control, as no signs of abnormalities in the trabecular structure of the bone could be found (Fig. 4B, lower panel).

## Discussion

3D printing is a fast and cost-effective method for the precise manufacture of products based on the experimental needs. Further, 3D printing has been used already to build human and animal-specific restrainers for a broad application in preclinical and clinical use [9–12]. The combination of sufficient protection and precise irradiation of target areas is essential for carrying out standardized radiation protocols with limited out-of-field systemic effects [13]. Salvarej et al. fabricated a small animal irradiation device for subcutaneous tumors which can restrain up to five mice while a lead plate ensures proper shielding effectiveness [6]. Lead was also chosen by Hasan et al. to build a shielding device for a gamma-cell chamber that allows irradiation of particular areas of mice [8]. Since lead material is generally a non-ecological friendly material with toxic characteristics, we evaluated different 3D printable filaments in respect to their X-ray shielding properties. 3D-printed tungsten filament, with its atomic number of 74 in the periodic table being slightly lower than of lead 82, showed the best shielding results in two different low-dose 3.5 Gy protocols we performed. Interestingly, tungsten material has been already evaluated as part of shielding clothes for low-dose exposed medical staff to replace heavy lead material [14]. Further, in nuclear medicine, tungsten materials have shown promising results as a replacement for lead as shielding material [15, 16]. The objective of our study was the design and evaluation of a 3D printable back shield for tumor-site specific irradiation of xenograft mice. We evaluated the custom-made shield by the use of a low-dose multible dosing protocol that was carried out four times at 3.5 Gy in a xenograft mouse model.

We could not find acute X-ray-induced toxicity in blood samples, spleens and bones of xenograft control/irradiation cohorts at the end of the experiments. Slightly but not significantly increased WBC counts of the xenograft control and irradiation groups are in the range of untreated mice but might be the result of the immunogenic stimulation by the injected tumor cells. Indeed, the increase in WBC counts is predominantly mediated by an increase of neutrophils in Xenograft control and irradiation groups. This increase has been reported in line with WBC stimulating effects which are mediated by the tumor cells itself [17]. Importantly, this increase in WBC counts was not attenuated by tumor site-specific X-ray irradiation in the xenograft irradiation cohort. Further, RBC counts were not significantly altered among the groups tested. Accordingly, there was no significant difference in spleen weights. These results suggest that good shielding of the rest of the animal’s body has taken place, as negative effects on spleen and blood cells can be observed even at low doses and single or repeated whole-body irradiation in mice [18, 19], which we decided not to repeat in the present experiments due to animal welfare considerations. The results of the blood analysis were further extended by the histological assessment of the spleen and the lemur bones. The spleen microarchitecture was not conspicuously altered in any of the groups which is in line with reports showing that low-dose X-ray irradiation enhances immune factors and does not per se alter white pulpa in mice [20]. Since it is known that radiation can have a negative effect on the bone marrow and bone structure [21], we also investigated the shielding properties of our back shield in this respect. The examination of the femurs of all groups investigated also provided no indication of radiation-induced damage, nor was the trabecular structure of the bone altered in any of the groups. The results of the histological investigation are consistent with the data obtained by blood sample analysis pointing towards adequate shielding of the animals by the tungsten filament printed back shield.

Following the in vivo experiments and histological examinations, we also evaluated the shields and molds with regard to handling properties in a laboratory animal facility. The designed X-ray shield is suitable for targeted irradiation of tumors in xenograft mouse models and can be used with various cabinet irradiation devices. Additional site openings of the shield with various sizes can be made upon necessity for localized irradiation of more than just one tumor site. The dimensions of the back shield can be individually adapted in the Shapr3D software to perfectly fit to the animal’s body size or respective tumor growth. Another advantage of the pie-shaped design is the placement of the animal in an almost physiological body position, which allows normal breathing in an upright position and does not require any further aids for fixation of limbs or rotation of the body. The simple pie-shaped design of the molds with integrated plug-in connectors allows the animals to be quickly transferred to the irradiation unit in a group of up to 8 animals. While the wing plates prevent the cover to put too much weight onto the animal, the top and back openings are required to ensure sufficient ventilation during the experiment. The tight compression of the filaments during the printing process ensures an almost smooth surface which can be fumigated or disinfected with e.g., alcohols which allows the use in specified-pathogen-free animal facilities.

## Conclusion

In this work, we showed that 3D-printed custom-designed back covers made of X-ray shielding tungsten filament can adequately protect the animal’s body in a low-dose irradiation protocol of xenograft mice. We present a fast, cost-effective and user-friendly device that meets high animal welfare standards.

## Data Availability

The print templates described in this manuscript are available for download on the thingiverse platform. Link: https://www.thingiverse.com/thing:6652646.

## Abbreviations

3D: Three dimensional
Gy: Gray
PLA: Polylactic acid
RBC: Red blood cell
WBC: White blood cell
